# Selection of internal reference gene for normalization of reverse transcription-quantitative polymerase chain reaction analysis in *Mycoplasma hyopneumoniae*

**DOI:** 10.3389/fvets.2022.934907

**Published:** 2022-07-22

**Authors:** Shiyang Li, Yanqing Zhou, Ting Yuan, Zhixin Feng, Zhenzhen Zhang, Yuzi Wu, Qingyun Xie, Jia Wang, Quan Li, Zhibang Deng, Yanfei Yu, Xiaomin Yuan

**Affiliations:** ^1^College of Veterinary Medicine, Hunan Agricultural University, Changsha, China; ^2^Key Laboratory of Veterinary Biological Engineering and Technology, Institute of Veterinary Medicine, Jiangsu Academy of Agricultural Sciences, Ministry of Agriculture and Rural Affairs, Nanjing, China; ^3^College of Veterinary Medicine, Nanjing Agricultural University, Nanjing, China; ^4^Zhongshan Institute for Drug Discovery, Shanghai Institute of Materia Medica, Chinese Academy of Sciences, Zhongshan, China; ^5^College of Veterinary Medicine, Yangzhou University, Yangzhou, China; ^6^School of Food and Biological Engineering, Jiangsu University, Zhenjiang, China

**Keywords:** *Mycoplasma hyopneumoniae*, RT-qPCR – real-time quantitative polymerase chain reaction, internal reference genes, gatB, virulence

## Abstract

*Mycoplasma hyopneumoniae* is the etiological agent of swine enzootic pneumonia (EP), which resulting in considerable economic losses in pig farming globally. Reverse transcription-quantitative polymerase chain reaction (RT-qPCR) is a major tool for gene expression studies. However, no internal reference genes for normalization of RT-qPCR data of *M. hyopneumoniae* have been reported. The aim of this study was to screen the most stable genes for RT-qPCR analysis in *M. hyopneumoniae* under different conditions. Therefore, a total of 13 candidate internal reference genes (*rpoC, Lipo, sgaB, oppB, hypo621, oppF, gyrB, uvrA, P146, prfA, proS, gatB*, and *hypo499*) of *M. hyopneumoniae* filtered according to the reported quantitative proteomic analysis and the *16S rRNA* internal reference gene frequently used in other bacteria were selected for RT-qPCR analysis. The mRNAs from different virulence strains (168, 168 L, J, NJ, and LH) at five different growth phases were extracted. The corresponding cycle threshold (Ct) values of the 25 reverse transcribed cDNAs using the 14 candidate genes were determined. Different internal reference genes or combinations were then screened for expression stability analysis using various statistical tools and algorithms, including geNorm, BestKeeper, and NormFinder software, to ensure the reliability of the analysis. Through further comprehensive evaluation of the RefFinder software, it is concluded that the *gatB* gene was the most suitable internal reference gene for samples of the different virulence strains in different growth phases for *M. hyopneumoniae*, followed by *prfA, hypo499*, and *gyrB*.

## Introduction

*Mycoplasma hyopneumoniae* is the primary pathogen of enzootic pneumonia (EP), a chronic respiratory disease in pigs, and one of the primary agents involved in the porcine respiratory disease complex (PRDC) (1). Studies of virulence factors have mainly focused on adhesion proteins, such as P97 and P102 (2, 3). However, the pathogenic mechanism is still unclear and additional virulence factors need to be discovered. These discoveries will be aided by reverse transcription-quantitative polymerase chain reaction (RT-qPCR). RT-qPCR is frequently used to screen genes with different expression levels in different samples (4). The difference in expression level of genes under specific conditions can be linked to specific phenotypes. For instance, genes with significantly different expression levels in strains with different virulence strains can be used to correlate the gene products with the virulence of the strains. However, a credible RT-qPCR is not available for *M. hyopneumoniae*. The key to the establishment of a reliable RT-qPCR system is the identification of internal reference genes. The inclusion of reference genes enables the normalization of sample-to-sample variation and avoids misinterpretation of the RT-qPCR assays. An ideal internal reference gene should be expressed consistently under various conditions. However, despite many studies, no internal reference gene capable of stable expression under all test conditions has been identified. The expression of internal reference gene changes in different types of cells and during different stages of cell growth (5). Hence, it is vital to identify the stable internal reference gene under particular conditions used in a study to allow accurate interpretation of the results. Many reports have described the screening of internal reference genes under different conditions. Previous experimental analyses determined that *rec*A, *rho, pro*C, and *rpo*D are the most appropriate reference genes for the normalization of RT-qPCR data in *Klebsiella pneumoniae* (6). Analysis of the weighted average covariance and NormFinder stability index of the expression of 39 genes implicated *mdo*G as a stable internal reference gene in *Escherichia coli* K-12 (7). A study in *Streptococcus thermophilus* showed that, compared with traditional RG *16S rRNA*, genes encoding glycine-tRNA ligase subunit β GlyS and fatty acid-binding protein DegV were more stably expressed (8). However, no internal reference genes of *M. hyopneumoniae* have been reported. This has prevented data normalization from this bacterium, which has hindered scientific gene transcription analysis and the comparison of results from different laboratories.

In this study, mRNAs extracted from *M. hyopneumoniae* strains in different growth phases and with diverse virulence were reverse transcribed into cDNAs and subjected to gene expression stability analysis by various statistical tools and algorithms, including geNorm, NormFinder, BestKeeper, and RefFinder.

## Materials and methods

### Strains and cultivation

High virulence *M. hyopneumoniae* strains 168, LH, and NJ and low virulence strains 168 L and J were cultured in modified Friis broth, KM2 medium (pH 7.4), at 37°C until the red medium turned yellow (pH 6.8). Details of the strains were mentioned in Data Sheet 1. Bacteria were harvested by centrifugation at 12,000 × *g* for 20 min at 4°C after culture for 12, 24, 36, 48, and 60 h. The pellets were washed twice with phosphate-buffered saline (PBS) before preparation for RNA extraction.

### Extraction of total RNA and reverse transcription

The extraction of RNA from a total of 25 samples of different strains at different growth stages was performed using the RNAprep Pure Cell/Bacteria Kit (TIANGEN BIOTECH CO., LTD., Beijing, China), followed by the manufacturer's instructions. First-strand cDNA synthesis was performed using a HiScriptIII Reverse Transcriptase Kit (Vazyme Biotech Co., Ltd., Nanjing, China), followed by the manufacturer's instructions. All cDNA samples were stored at −80°C until used. The details of the reverse transcription reaction system are summarized in Table 1.

**Table 1:** Reverse transcription reaction system details.

**Reagent**	**Volume**
5 × HiScript II Buffer	4 μL
dNTP Mix (10 mM each)	1 μL
HiScript II Reverse Transcriptase (200 U/μl)	1 μL
RNase inhibitor (40 U/μl)	1 μL
Oligo (dT) 23VN (50 μM)	1 μL
Random hexamers (50 ng/μl)	1 μL
Total RNA	100 ng
RNase-free distilled deionized water	up to 20 μL

### Design and validation of specific primers

The candidate reference genes were chosen according to the proteomic analysis of *M. hyopneumoniae* (9). Genes displaying constant expression levels between the two strains with different virulence strains were used for further analysis of transcript levels. The threshold was defined with the abundance fold change between −1.1 and 1.1, because fold change > 1.2 (10), 1.5 (11), or 2.0 (12) was considered significant in most comparative proteomics analyses. According to the standards, a total of 13 candidates were chosen. These included DNA-directed RNA polymerase subunit beta (*rpoC*), lipoprotein (*Lipo*), pentitol phosphotransferase enzyme II, B component (*sgaB*), oligopeptide transport system permease protein (*oppB*), hypothetical protein (*hypo621*), oligopeptide transport system permease protein (*oppF*), DNA gyrase subunit B (*gyrB*), excinuclease ABC subunit A (*uvrA*), *P146* adhesin like-protein, p97 paralog (*P146*), peptide chain release factor 1 (*prfA*), prolyl-tRNA synthetase (*proS*), glutamyl-tRNA amidotransferase subunit B (*gatB*), and hypothetical protein (*hypo499*). In addition, another candidate commonly used in other bacteria, 16S ribosomal RNA (*16S*), was also evaluated in this study. *16S* is a housekeeping gene among different strains and its transcription level is reported to be relatively stable (13). Specific primers of the 14 candidates (Table 2) were synthesized by GenScript Biotech (Nanjing, China).

**Table 2:** RT-qPCR primers for candidate reference genes.

**Gene**	**Forward primer (5'-3') Reverse primer (5-3')**	**Length of products, bp**
*16S*	F: CGGCAGTATCTTTAGGGTTCTC R: GCTCGTGTCGTGAGATGTTAG	95
*gyrB*	F: AAACGCCCGGGAATGTATATC R: CTGCAAGAGCCTCATCAACT	97
*gatB*	F: AATGGATCACTTCGTGCTGATA R: TCAAGTTCGGCGGCTTT	125
*hypo499*	F: CATAGGAAGGCAAGCCTCAA R: CGATGAGGCAACTAGGGTAATAG	110
*hypo621*	F: CGCGAGTGCTGATCGTATTT R: AGATGGCGGTGATCTTTCTTG	135
*Lipo*	F: GCAAGTTGTTGGGAGGTAATTG R: AAGAAATCGCTGAGGGTAGTG	133
*oppB*	F: TCTATTCCCGGAGATCCAAGT R: AACGTTGTGCTTGTGGTAAATC	100
*oppF*	F: CTGTCTTTGACCACCGGAAA R: GAAGCACTTGAAAGCGTCAATC	78
*P146*	F: GAGGGTGAGGAAGATGAAGAAG R: GAAGTCAACTCCAAGACGAAGA	100
*prfA*	F: GAATTCGGCCCATTGTTTCAG R: CTTTAGCAGGCGGGTTTAGT	129
*proS*	F: CTCCCGAAAGAGAGCAAGAAA R: CCTGTCTGTGGTAAGGCAAA	94
*rpoC*	F: GGCCTTCTTTGGTTGTTACTTG R: CCGCTCATGCTCCTGTTATTA	117
*sgaB*	F: GCTGCTTGTGGAAATGGAATG R: CGCTTCAACTGTGGCATCTA	96
*uvrA*	F: GTAGGACATCCGGCTTGTATC R: CCAAGGAGACGGGCAAATTA	101

To evaluate the specificity of the candidate internal reference genes, both agarose gel electrophoresis and melting curves of primers analysis were performed. Using the strain cDNA as a template, the RT-qPCR amplification products of 14 internal reference genes were verified by 1% agarose gel electrophoresis to investigate the homogeneity and purity of the products, evident as a single band. The melting curves of the primer analyses determined using the QuantStudio 5 software were also analyzed to determine if the dissolution peaks were single and sharp. A sharp peak will appear in the curve if the reaction product is single. At least two or more peaks will appear if there is a dimer or non-specific amplification.

### RT-qPCR analysis

Taq Pro Universal SYBR qPCR Master Mix was used for RT-qPCR analysis (Vazyme Biotech Co., Ltd.). All tests were carried out in biological triplicates and technical duplicates. The experimental reaction system conditions are presented in Table 3. RT-qPCR was performed for each candidate reference gene according to the procedures listed in Table 4. The cycle threshold (Ct) values of 25 samples were collected for statistical analyses.

**Table 3:** RT-qPCR reaction system details.

**Reagent**	**Volume**
2 × Taq PRO Universal SYBR qPCR Master Mix	10.0 μL
Primer F	0.4 μL
Primer R	0.4 μL
Template cDNA	2 μL
ddH2O	8 μL

**Table 4:** RT-qPCR reaction procedures.

**Item**	**Reps**	**Temperature**	**Time**
Predenaturation	1	95 °C	30 s
Cyclic response	40	95 °C	10 s
		60 °C	30 s
		95 °C	15 s
Dissolution curve	1	60 °C	60 s
		95 °C	15 s

### Expression stability analysis

The stability of gene expression level was analyzed using the geNorm (14, 15), NormFinder (16, 17), and BestKeeper software (18, 19).

#### GeNorm

The data analysis was first performed using geNorm (14). The Ct value of 25 samples was converted into a relative quantitative Q-value using the following formula: Q = 2–^ΔCt^ (ΔCt=Ct sample–Ct min). “Ct sample” was the Ct value of the housekeeping gene in each of the samples of *M. hyopneumoniae* with different virulence strains and in different growth stages. “Ct min” indicated the lowest Ct value of the housekeeping genes among the 25 samples. Then, the expression stability measurement (M) value was calculated with Q-value by the geNorm program for each candidate reference gene. The optimal number of reference genes was determined by the paired coefficient of variation Vn/Vn+1. When Vn/V (n + 1) <0.15, the optimal number of internal reference genes is n. When Vn/V (n + 1) > 0.15, the optimal number of internal reference genes is n + 1. GeNorm did not differentiate between groups of samples or treatments.

#### NormFinder

The principle of NormFinder was similar to that of the geNorm program. The stable value of gene expression was the same as the M-value of geNorm (16). However, NormFinder offered a method of reference gene selection that took into account intragroup and intergroup variability. The gene with the smallest stable value of expression was taken as the most stable. Another characteristic of the NormFinder software was that only one appropriate internal reference gene would be selected as the reference gene.

#### BestKeeper

BestKeeper is also a program designed for selection against internal reference genes (19). The raw data were filled into the table of the BestKeeper software. The expression stability was evaluated by calculating standard deviation (SD) and percentage covariance (CV). Both BestKeeper and geNorm were based on pairwise comparison, which carried the same vulnerabilities regarding co-regulated genes (20). Internal reference genes and target genes were analyzed separately in this program. The BestKeeper program produces paired correlation coefficients and BestKeeper indices (geometric mean of Ct values of each candidate gene) between genes, which are compared according to the magnitude of their values.

### Integrated analysis by RefFinder

Finally, the results obtained from geNorm (M-values), NormFinder (stability values), and BestKeeper (CV and SD) were subjected to the RefFinder algorithm (http://150.216.56.64/referencegene.php?type=reference), which integrated the results of the aforementioned three standard analysis algorithms for the comprehensive ranking of candidate reference gene(s) (21).

## Results

### Validation of specificity of candidate internal reference genes

**Figure 1:**
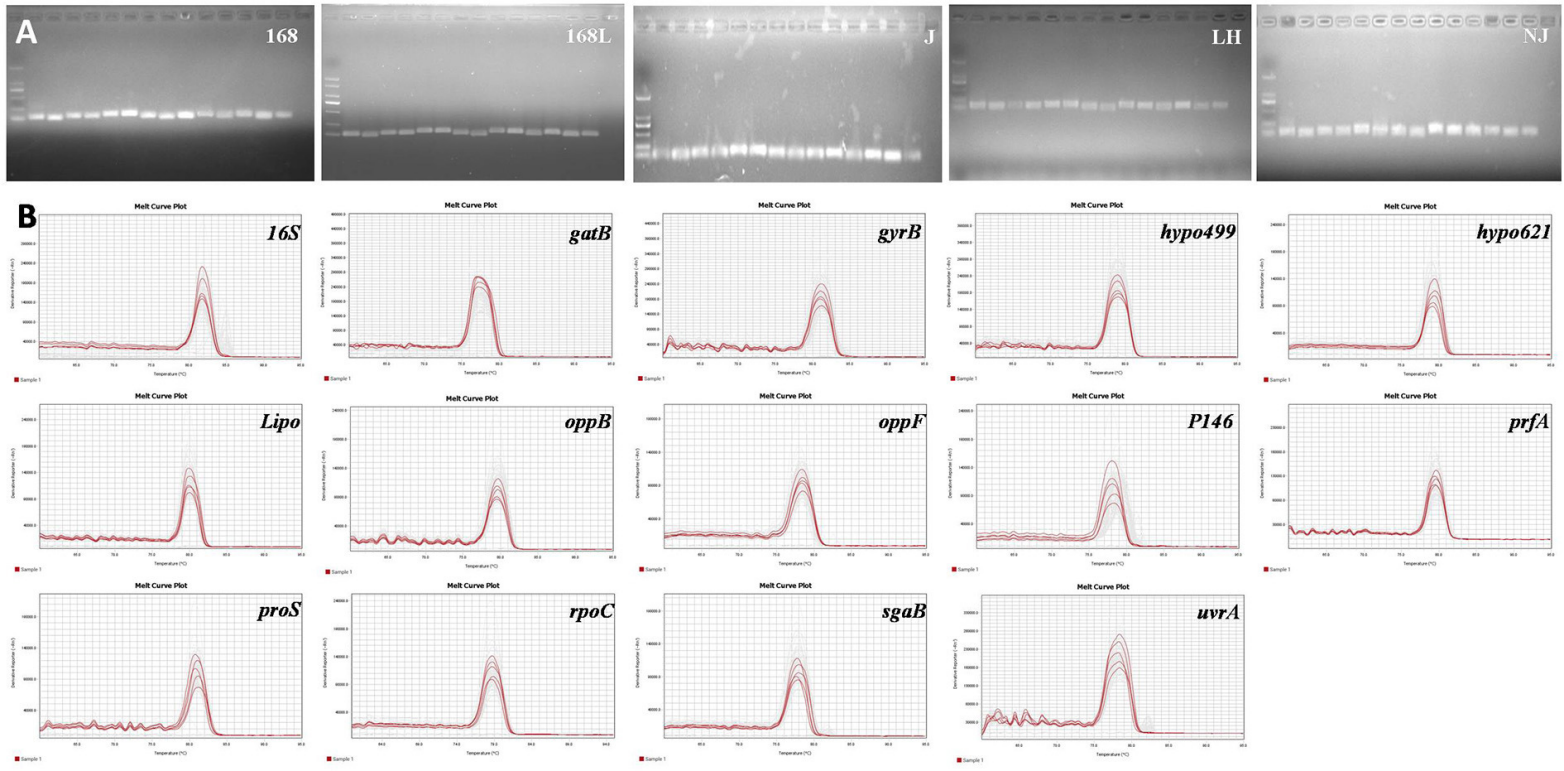
**(A)**. Nucleic acid gel electrophoresis of candidate reference genes. M: DL2000;1: *16S*; 2: *gyrB*; 3: *gatB*; 4: *hypo499*; 5: *hypo621*; 6: *Lipo*; 7: *oppB*; 8: *oppF*; 9: *P146*; 10: *prfA*; 11: *proS*; 12: *rpoC*; 13: *sgaB*; 14: *uvrA*. **(B)**. Melting curves of the 14 candidate reference genes using genomes of different *M. hyopneumoniae* strains. Melting temperatures were visualized by plotting the negative first derivative of fluorescence relative to temperature (°C).

The expression stability of housekeeping genes is the determining factor for the screening of internal reference genes. According to this principle, the genes previously reported to be expressed constantly in two different virulence strains (9), as well as the frequently used internal reference target *16S*, were chosen for further evaluation. Prior to the RT-qPCR assay, the validity of the designed primers for a total of 14 candidates was subjected to reference genes specificity evaluation using cDNA transcribed from RNA as the template. RNA of 25 different samples from *M. hyopneumoniae* strains with different virulence strains and in different growth stages was obtained by RNA extraction. Equal amounts of RNA were further used as the template for the reverse transcription reaction to obtain cDNA. The obtained RT-qPCR amplification products of 14 internal reference genes using cDNA of 5 different strains as the template were verified by 1% agarose gel electrophoresis. As shown in Figure 1A, clear and specific target bands were obtained from all 14 tested internal reference genes using the genomes of five different strains as a template. In addition, the good specificity of the 14 primers was also validated by the melting curve analysis. As shown in Figure 1B, the melting curves of all the 14 primers exhibited a single and sharp peak in 5 different stains, which confirmed the specificity of every primer pair.

### Abundance of transcripts of candidate internal reference genes

**Figure 2:**
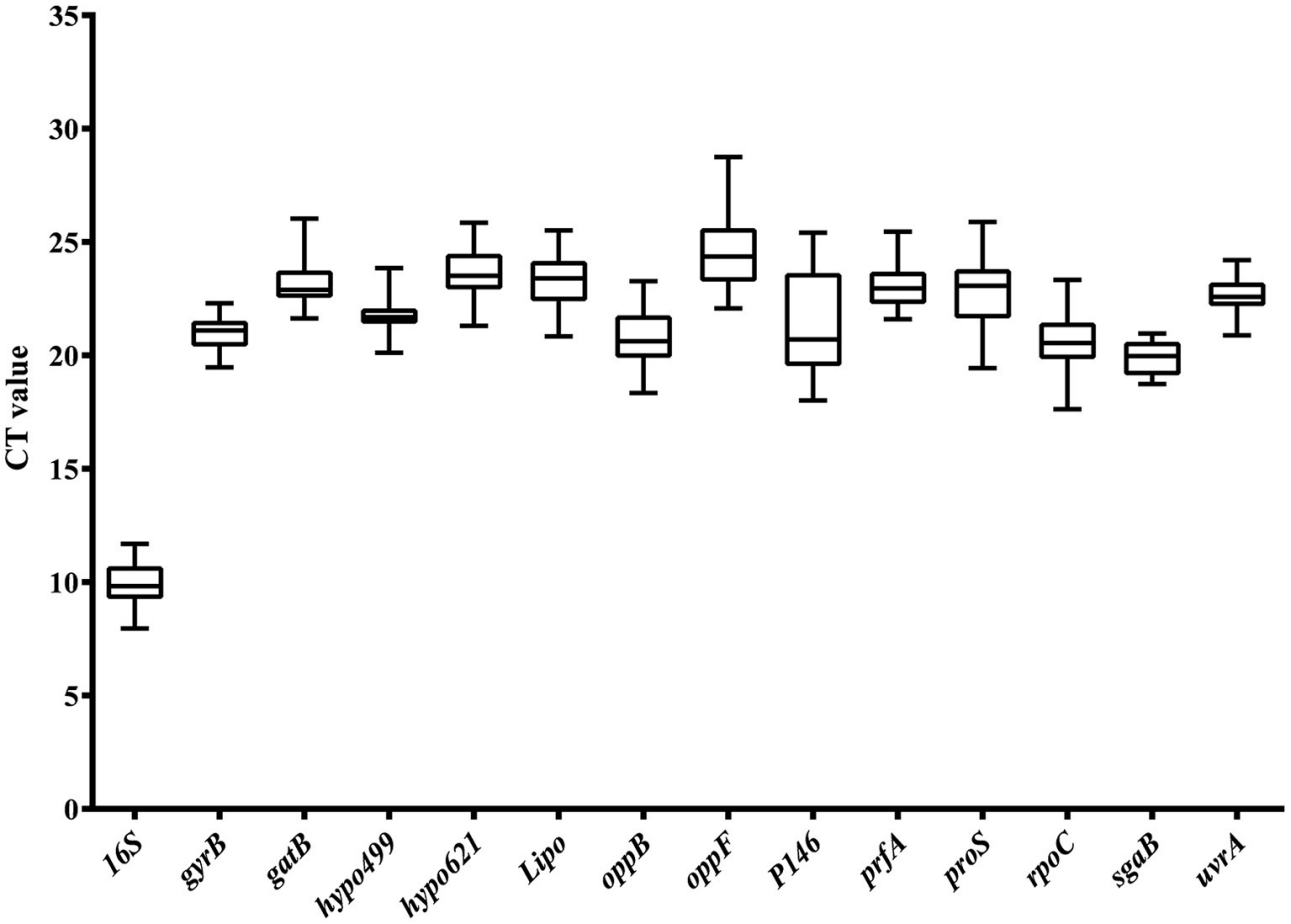
Ct value distribution of candidate reference genes in all samples by RT-qPCR. Boxes represent the mean Ct value and bars depict the mean ± standard deviation.

Analyzing the abundance of mRNA level was another important prerequisite for the selection of internal reference genes. A moderate level of gene expression was an optimal choice for reference genes, because it allows the evaluation of genes expressed at various levels, including very high and very low. The Ct value of reference genes was inversely proportional to the expression level of the genes in RT-qPCR analysis. The greater the Ct value of the reference gene, the lower the expression of the target gene in the sample and vice versa. Expression abundance of the 14 candidate reference genes in all 25 samples was analyzed using serial 10-fold dilutions of PCR products *via* RT-qPCR. The Ct values for each reference gene ranged from 8 to 29 (Figure 2). The large distribution of Ct values suggested that the expression abundance differed among the reference genes. The minimum Ct value of 8 was displayed by *16S*. Thus, the gene expression abundance of *16S* was the highest. The extremely high abundance of the reference genes will hinder the evaluation of the transcriptional level of those genes with low abundance. Thus, *16S* was excluded from the candidate reference genes pool in the following analysis. The Ct values of the other 13 genes were approximately 21, which was neither too high nor too low, and suitable to evaluate the transcription levels of other genes.

### Stability of candidate internal reference genes

The transcription stability is vital for the internal reference of the 13 housekeeping genes with moderate abundance. This stability was determined for all 25 *M. hyopneumoniae* samples in different growth phases and with different virulence strains.

#### GeNorm analysi

**Figure 3:**
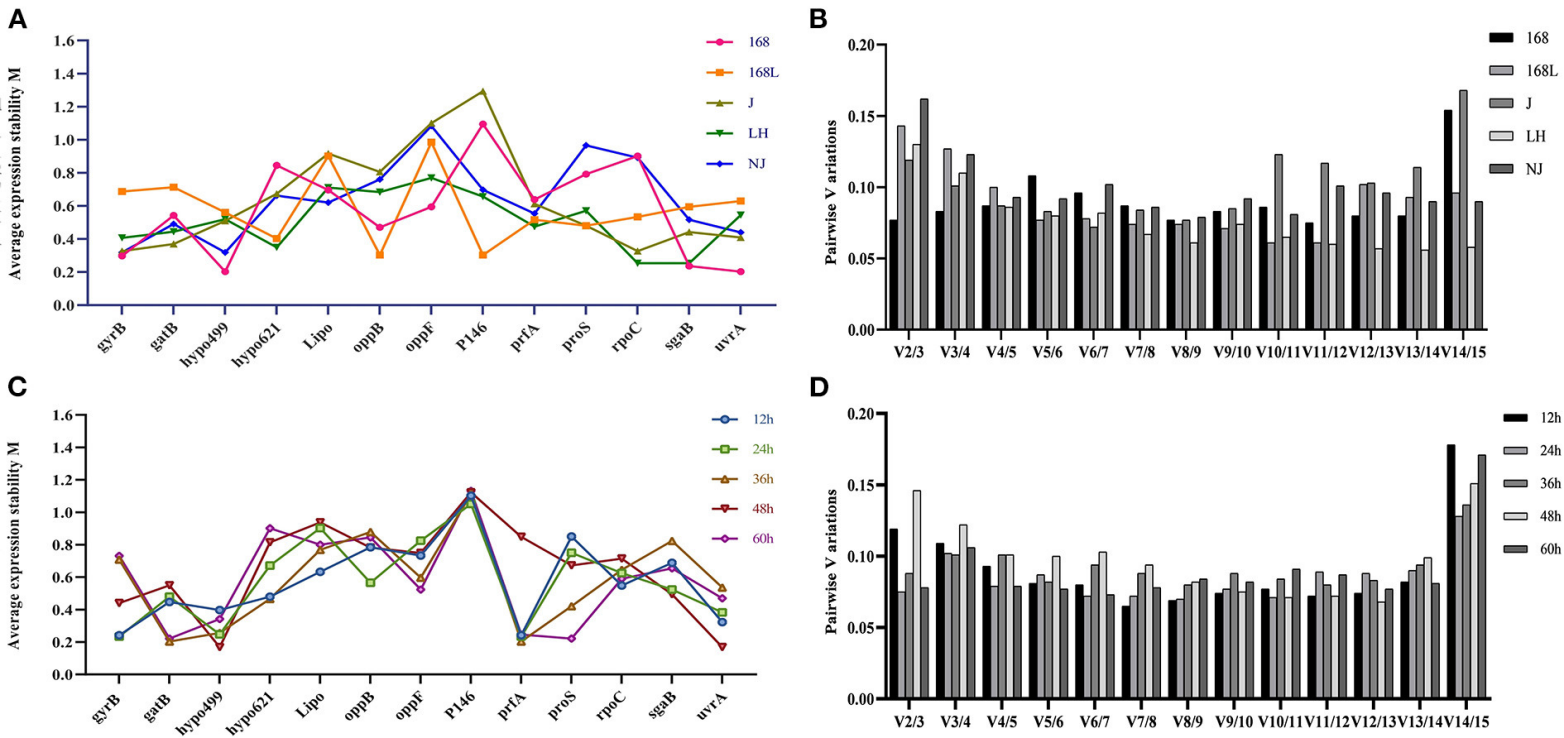
Average expression stability M-value and optimal number of reference genes according to geNorm analysis. The expression stability of 13 candidate genes in the same strain at different culture time **(A)** and different strains at the same culture time **(C)** was calculated. The x-axis represents various candidate reference genes. The y-axis represents stability value (M-value). Lower M-value suggests higher expression stability. **(B)** and **(D)** show the optimal number of reference genes in different subsets. The x-axis represents the number of genes selected for comprehensive analysis V (n/n+1), and the y-axis means the pairwise variation value (V-value). When the V-value is <0.15, the corresponding combination is deemed stable; n is the best number of internal reference genes.

All 25 *M. hyopneumoniae* samples were split into two sets according to the same strain at different growth stages and different virulence strains at the same growth stages, which were entered separately in the geNorm software package. Figures 3A,C shows the geNorm M analysis for all samples. Lower geNorm M values represented more stable reference genes. In addition, it is generally considered that when the value of Vn/V (n+1) calculated with Ct values of different primers and different samples by geNorm is <0.15, it is unnecessary to introduce a new reference gene into the internal reference system. Otherwise, (n+1)^th^ reference gene is needed. As shown in Figure 3B, the V2/3 values of reference genes in all samples, except for strain NJ, were less than the threshold value of 0.15. Gene expression analysis by geNorm recommended two reference genes to achieve the best performance for strains 168, 168 L, J, and LH, while 3 reference genes were suitable for strain NJ. Results shown in Figure 3A demonstrated that for strain 168 at different growth stages, the candidate *hypo499* and *uvrA* genes had the lowest M-values and were ranked as the most stable candidates. Accordingly, the candidate *oppB* and *P146* genes ranked as the most stable genes in strain 168 L at different growth stages. In strain J, the stability of *gyrB* and *rpoC* was highest. For strain LH, the targets of highest transcriptional stability in different culture times were *rpoC* and *sgaB*. For strain NJ, *gyrB, hypo499*, and *uvrA* were the most stable.

The stability of internal reference genes for samples in the same growth phases of different strains was further evaluated. As shown in Figure 3D, two reference genes were suitable for RT-qPCR normalization in 12-h, 24-h, 36-h, 48-h, and 60-h cultures of different strains. The most stable candidates of 12-h cultures of the above five different strains were *gyrB* and *prfA*. Genes *gyrB* and *prfA* ranked as the two most stable candidates due to their minimum M-values. *P146* and *prfA* similarly ranked as the two most stable genes of 24-h cultures of the different strains. The most stable reference genes of 36-h cultures of the different strains were *gatB* and *prfA*. Genes *hypo499* and *uvrA* were the two most stable genes of 48-h cultures of the different strains. The candidate reference genes with maximum stability of 60-h cultures of the different strains were *gatB* and *proS*.

#### NormFinder analysis

**Figure 4:**
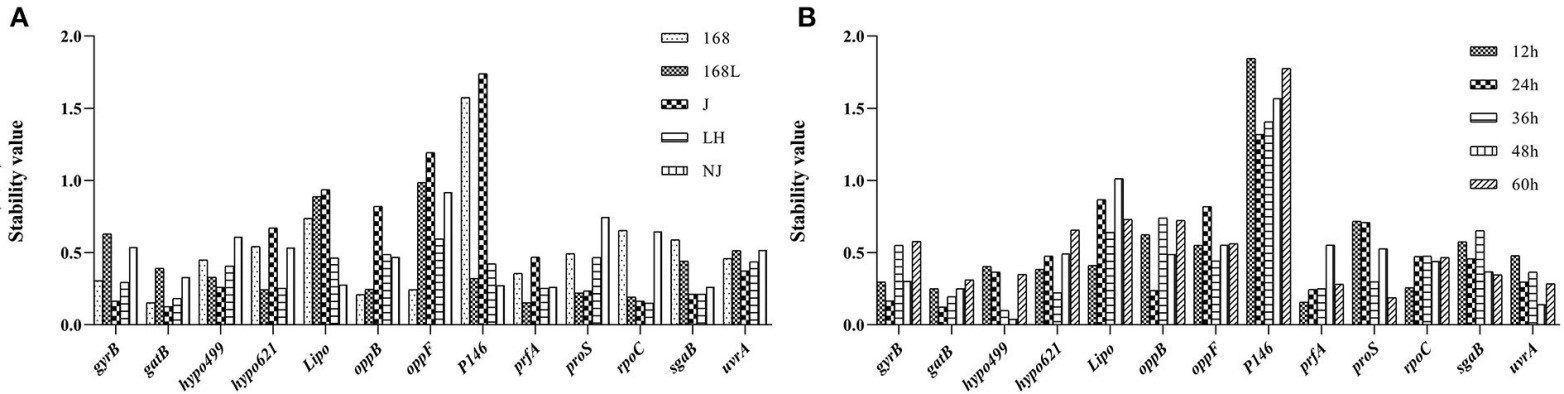
Stability analysis of internal reference genes using the NormFinder software. The x-axis represents various candidate reference genes. The y-axis represents stability value. **(A)**: stability value of reference gene of the same strain at different culture time. **(B)**: stability value of reference gene of different strains at the same culture time.

NormFinder was a similar software to geNorm in the calculation method. The lower stability value calculated from NormFinder indicated the higher stability of the reference gene expression. The difference in NormFinder with geNorm was that NormFinder selected only one most suitable candidate internal reference gene. *GatB* was shown to be the most stable reference gene to evaluate the gene expression level in strains 168 and J at different growth phases (Figure 4A). *PrfA* ranked as the most stable reference gene at different growth stages of strains 168 L and NJ. *RpoC* ranked as the most stable reference gene for strain LH under different culture times.

In the samples of different strains with a fixed culture time of 12 h, *prfA* displayed the highest stability (Figure 4B). The gene with the highest stability in 24-h cultures of the five different strains was *gatB*. *Hypo499* displayed the highest stability in both 36-h and 48-h cultures among all five different strains. *ProS* was the most potential candidate for the evaluation of the gene expression changes in 60-h cultures of different strains.

For strain LH at different growth phases, the top two genes analyzed by geNorm analysis included the most stable gene *rpoC* obtained from the NormFinder algorithm. In the samples of different strains with fixed culture times of 12 h, 48 h, and 60 h, the respective most stable genes, *prfA, hypo499*, and *proS*, from NormFinder analysis ranked top two in geNorm.

#### BestKeeper analysis

**Figure 5:**
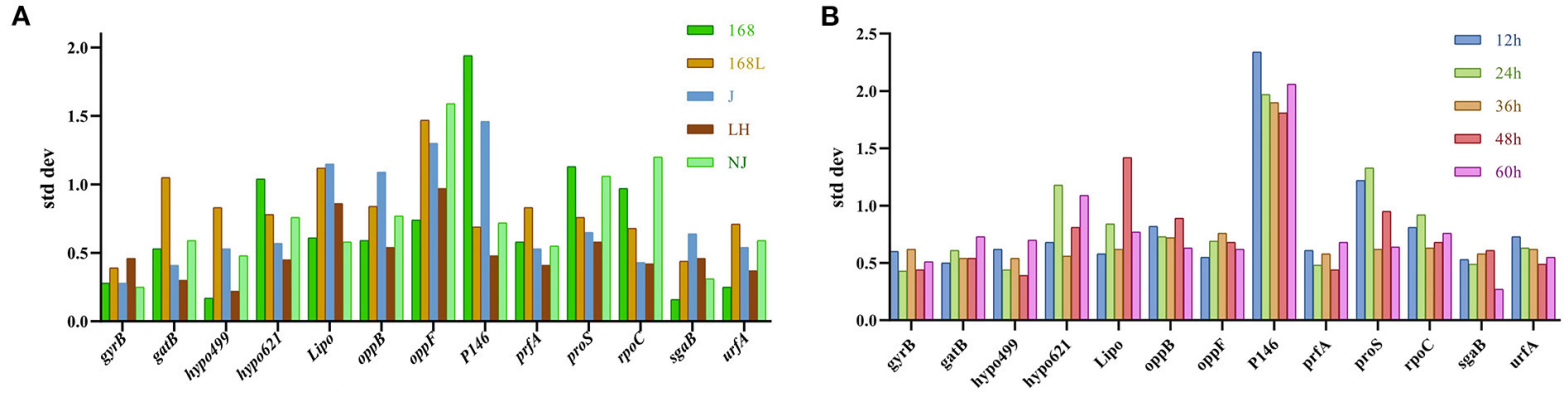
Analysis of internal reference gene standard deviation (SD) using the BestKeeper software. The x-axis represents various candidate reference genes. The y-axis represents stability value. **(A)**: SD value of reference gene of the same strain at different culture times. **(B)**: SD value of reference gene of different strains at the same culture time.

BestKeeper was also used to determine the expression stability of the candidate reference genes by calculating the SD and CV of the Ct values obtained from samples of different stains in different growth phases. The lower the SD value, the higher the gene stability. Genes with SD value >1 were considered unsuitable as reference genes (22). As shown in Figure 5A, the *sgaB* gene was most stable in strain 168 in different growth stages. *Hypo499* was most stable in strain LH harvested at different times. *GyrB* was the most stable reference gene in strains 168 L, J, and NJ harvested at different times.

The *gatB* gene was the most stable reference gene in all strain samples cultured for 12 or 36 h (Figure 5B). For 24, 48, and 60 h cultures, *gyrB, hypo499*, and *sgaB* were the most stable genes, respectively. The most unstable internal reference gene among the 13 candidates at different culture times was *P146*, which was the same as the results obtained from both geNorm and NormFinder.

For strains J and NJ at different growth phases, the most stable gene *gyrB* analyzed by BestKeeper analysis was included in the top two genes obtained from the geNorm algorithm. In the samples of different strains with fixed culture times of 36 h and 48 h, the most stable gene *hypo499* from BestKeeper analysis ranked top one in NormFinder.

#### Integrated analysis by RefFinder

**Figure 6:**
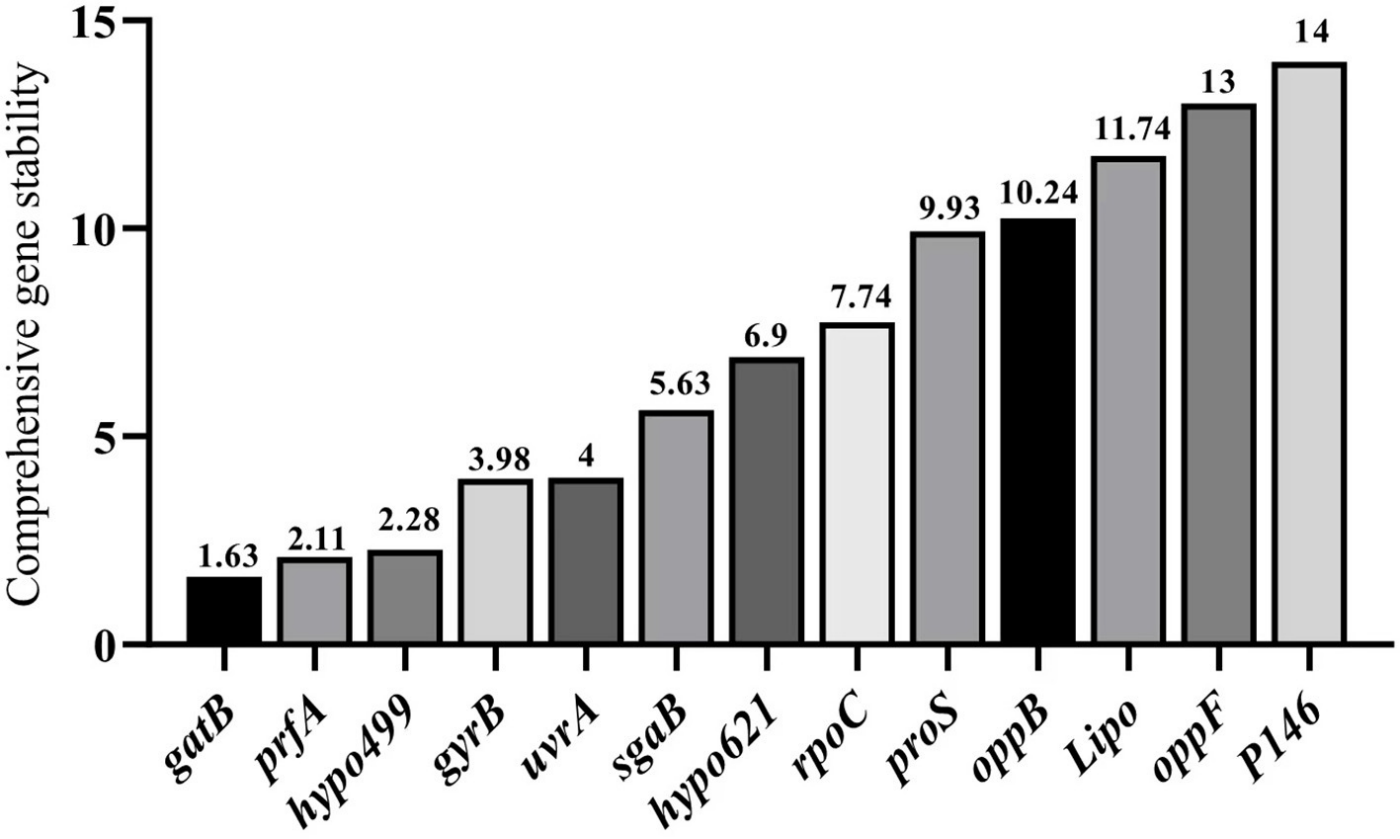
RefFinder analysis of expression stability of candidate internal reference genes. The x-axis represents various candidate reference genes. The y-axis represents stability value.

RefFinder integrates all the results of the three aforementioned methods to calculate the geometric mean for each reference gene and their comprehensive ranking index of stability. A lower index value indicates a higher stability of the reference gene. The RefFinder comprehensive analysis displayed in Figure 6 showed that the expression stability of 13 internal reference genes from high to low under different culture time conditions of different strains of *M. hyopneumoniae* was: *gatB, prfA, hypo499, gyrB, urfA, sgaB, hypo621, rpoC, 16S, proS, oppB, Lipo, oppF*, and *P146*. In all samples, the top four most stable reference genes were *gatB, prfA, hypo499*, and *gyrB*, and the most unsuitable reference gene was *P146*, which was consistent with the results from geNorm and NormFinder (Figure 6). For simplicity of use, the most stable gene, *gatB*, can be used for gene expression analysis among different *M. hyopneumoniae* strains at different growth phases.

### Validation and application

**Figure 7:**
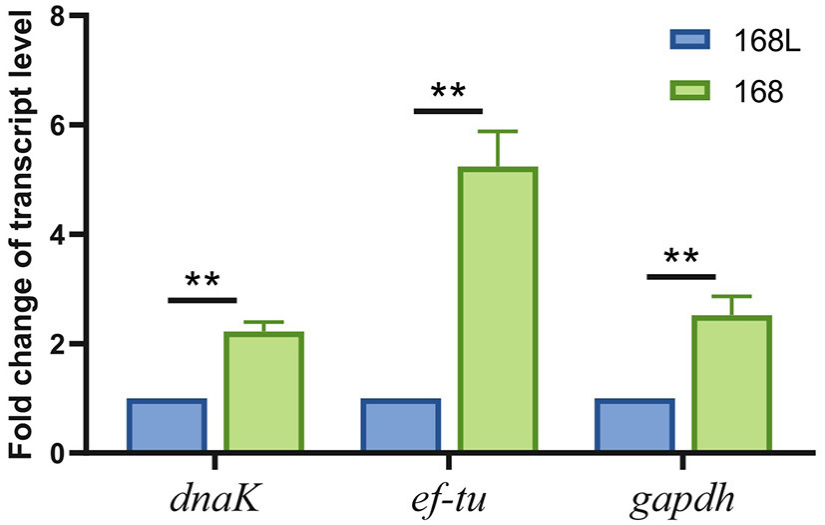
Transcription levels of *dnak, ef-tu*, and *gapdh* genes between high virulence *M. hyopneumoniae* strain 168 and low virulence strain 168 L.

To confirm the validity of using *gatB* as an internal reference gene for RT-qPCR analysis to screen the virulence-associated genes of *M. hyopneumoniae*, we evaluated the expression level of a reported virulence factor *ef-tu* in this study. We found that the abundance of *ef-tu* gene expression products in high virulence strain 168 was significantly higher than that in attenuated strain 168 L, which indicated the relevance of *ef-tu* with *M. hyopneumoniae* virulence (Figure 7). The results were consistent with the reported research, in which *ef-tu* encoding products were found to contribute to the adhesion process of *M. hyopneumoniae* (23). The consistency of the results using *gatB* as the internal reference gene in RT-qPCR and the reported data demonstrated the reliability of the established RT-qPCR method.

The established RT-qPCR method using *gatB* as the internal reference gene was then used to identify novel virulence-related genes. *Gapdh* and *dnaK* were found significantly different in transcript levels in high virulence strain 168 and low virulence strain 168 L. GAPDH of *Mycoplasma hyorhinis* was found to be an adhesin to epithelial cells as well as a plasminogen receptor mediating extracellular matrix (ECM) degradation (24). DnaK of *M. hyorhinis* functioned as a multi-binding protein on the surface of *M. hyorhinis* cells (25). GAPDH and DnaK of *M. hyopneumoniae* were also found to contribute to ECM degradation, which may help the pathogen break through the tissue barrier for further invasion (data unpublished). These findings demonstrated that the established RT-qPCR method can be used to discover novel phenotype (virulence/growth phases)-related genes.

## Discussion

Discovering physiological and pathological functions of key genes is of great significance for studies of pathogenic mechanism of *M. hyopneumoniae*. The relative quantification of gene expression is mainly realized through RT-qPCR. Suitable reference genes are necessary for the quantification of key gene expression patterns. To date, although no study on identifying reference genes of *M. hyopneumoniae* had been conducted, many studies on identifying suitable reference genes of other bacteria had been reported (26). As found in these studies, genes with the most stable expression patterns were always different in different species, at different developmental stages, or with different virulence strains, indicating the complexity of suitable reference genes (6, 27, 28). Therefore, it was necessary to conduct careful verification to identify the most stable reference genes in different *M. hyopneumoniae* strains with various virulence strains at different growth phases.

In this study, to identify the most stable reference gene, we used four statistical approaches to estimate the expression stability of 14 candidate reference genes in 5 different *M. hyopneumoniae* strains with various virulence strains and at 5 different growth phases. Various combinations of internal reference genes for RT-qPCR analysis of *M. hyopneumoniae* in different conditions were proposed. Although the optimal internal reference genes calculated by geNorm, NormFinder, BestKeeper, and ReFinder for *M. hyopneumoniae* RT-qPCR analysis between different virulence strains and under diverse growth phases were not completely identical; the rank of the most stable reference genes did not show great differences. Of the 14 candidate reference genes, *gatB, prfA, hypo499*, and *gyrB* were consistently among the top few optimal internal reference genes in all three methods, geNorm, NormFinder, and BestKeeper, respectively. Other studies also reported different ranking orders using different statistical approaches when identifying suitable reference genes under different developmental stages and temperature stresses (29). To comprehensively utilize the data analyzed by the three methods to obtain one best reference gene for the sake of simplicity, integrated analysis by ReFinder was further performed. The online software selected *gatB* as the comprehensive optimum reference gene for the analysis of gene expression differences of both different virulence strains and strains at different growth phases.

The internal reference genes screened in this study were then subjected to reevaluation with the reported virulence-associated genes in *M. hyopneumoniae*. The known virulence factor *ef-tu* (30) and novel virulence-associated genes *gapdh* (24) and *dnaK* (25) were also highly transcribed in virulence strains when RT-qPCR analysis was performed using *gatB* as an internal reference gene. This further validated the credibility of our screened internal reference genes.

In addition to virulence and growth cycles, the *gatB* internal reference genes may be used to assess gene expression levels in dynamic conditions, such as cultures in different media. However, more validation needs to be done. Meanwhile, there are also some limitations in the application of this method. It can certainly be used to evaluate changes in gene transcription levels. However, it cannot connect a specific gene to certain phenotypes, because in most cases proteins are the final functional form of the gene, and there are many ways of regulation from RNA to proteins.

## Data availability statement

The original contributions presented in the study are included in the article/supplementary material, further inquiries can be directed to the corresponding authors.

## Author contributions

SL performed most of the experiments. SL and YY prepared the manuscript. YZ and JW helped with the statistical analyses. TY and YW are responsible for the preparation of the experimental materials. ZZ, QX, and QL participated in the evaluation process. ZF, XY, and ZD supervised the work. All authors contributed to the article and approved the submitted version.

## Conflict of interest

The authors declare that the research was conducted in the absence of any commercial or financial relationships that could be construed as a potential conflict of interest.

## Publisher's note

All claims expressed in this article are solely those of the authors and do not necessarily represent those of their affiliated organizations, or those of the publisher, the editors and the reviewers. Any product that may be evaluated in this article, or claim that may be made by its manufacturer, is not guaranteed or endorsed by the publisher.
