# Supplementary material for: Selection of internal reference gene for normalization of reverse transcription-quantitative polymerase chain reaction analysis in Mycoplasma hyopneumoniae
Source: Front Vet Sci. 2022 Jul 22;9:934907. doi: 10.3389/fvets.2022.934907 (PMC9355380; doi:10.3389/fvets.2022.934907)
Supplement: Supplementary file 1 [file Data_Sheet_1.docx]

Supplementary table 1. A list of strain details taken in the study

| Strains | Date | Virulence | Geographical origins | Reference |
| --- | --- | --- | --- | --- |
| 168 | 1974 | high | China | [[1](#_ENREF_1)] |
| 168L | - | low | China | [[2](#_ENREF_2)] |
| NJ | 2004 | high | China | [[3](#_ENREF_3)] |
| J | - | low | Brazil | [[4](#_ENREF_4)] |
| LH | 2016 | high | China | - |

**Reference**

[1] Liu W., Feng Z.X., Fang L.R., Zhou Z.M., Li Q.A., Li S., Luo R., Wang L., Chen H.C., Shao G.Q., Xiao S.B., Complete Genome Sequence of Mycoplasma hyopneumoniae Strain 168, Journal of Bacteriology. (2011) 193:1016-+.

[2] Liu W., Xiao S.B., Li M., Guo S.H., Li S., Luo R., Feng Z.X., Li B., Zhou Z.M., Shao G.Q., Chen H.C., Fang L.R., Comparative genomic analyses of Mycoplasma hyopneumoniae pathogenic 168 strain and its high-passaged attenuated strain, BMC Genomics. (2013) 14.

[3] Wang H.Y., Zhang Z.Z., Xie X., Liu B.B., Wei Y.N., Gan Y., Yuan T., Ni B., Wang J., Zhang L., Xiong Q.Y., Shao G.Q., Feng Z.X., Paracellular Pathway-Mediated Mycoplasma hyopneumoniae Migration across Porcine Airway Epithelial Barrier under Air-Liquid Interface Conditions, Infection and Immunity. (2020) 88.

[4] Zielinski G.C., Ross R.F., Effect of Growth in Cell-Cultures and Strain on Virulence of Mycoplasma-Hyopneumoniae for Swine, American Journal of Veterinary Research. (1990) 51:344-348.
